# 1400. Emergency Department (ED) Use among Persons With HIV (PWH) before and during the COVID-19 Pandemic

**DOI:** 10.1093/ofid/ofac492.1229

**Published:** 2022-12-15

**Authors:** Deana Agil, Lindsay Browne, Thibaut Davy-Mendez, Amy Durr, Heather Henderson, Kuo-Ping Li, Claire E Farel, Joseph J Eron, Sonia Napravnik

**Affiliations:** UNC Chapel Hill, Chapel Hill, North Carolina; UNC Chapel Hill, Chapel Hill, North Carolina; UNC Chapel Hill, Chapel Hill, North Carolina; UNC Chapel Hill, Chapel Hill, North Carolina; UNC Chapel Hill, Chapel Hill, North Carolina; UNC Chapel Hill, Chapel Hill, North Carolina; UNC-Chapel Hill, Chapel Hill, North Carolina; University of North Carolina at Chapel Hill School of Medicine, Chapel Hill, North Carolina; UNC Chapel Hill, Chapel Hill, North Carolina

## Abstract

**Background:**

Substantial changes in access and delivery of primary HIV care occurred during the COVID-19 pandemic. To assess how care access changed during the COVID-19 pandemic, we estimated ED use among PWH in care 2017-2021 in the southeastern US.

**Methods:**

For each calendar year, among PWH in care in the UNC CFAR HIV Clinical Cohort (defined as having a clinic visit in the current or prior year), we estimated the percent of patients with ≥ 1 ED visit in a given year, overall and by age, gender, race/ethnicity, HIV viral load (VL), and CD4 count. We estimated risk ratios (RRs) comparing patient characteristics and years 2020-2021 vs. 2017-2019, using Poisson regression with generalized estimating equations to account for repeated measures.

**Results:**

Among 2129 PWH in care 2017-2021 (N≈1700-1800 in each year), 57% identified as Black, 31% White, 8% Hispanic, 26% women, with median age of 47 years (IQR 35-55). During the study period, there were 3645 ED visits over 8813 person-years, a rate of 41.4 ED visits-per 100 person-years(95% CI 36.8-46.5) per 100 person-years. The 845 PWH with at least one ED visit during the study period contributed a median of 2 visits each (IQR 1-5). The unadjusted probability of having ≥1 ED visit in a given year was higher among women vs. men (RR=1.14, 95% CI 0.99-1.32), Black vs. White PWH (1.31, 1.13-1.52), with VL ≥ 40 copies/mL (1.40, 1.20-1.64), and with CD4 < 200 (1.66, 1.32-2.09) or 200-349 (1.50, 1.25-1.79) vs. ≥ 500 cells/μL; age was not associated with ED use. Compared with 2017-2019, the annual probability of having ≥ 1 ED visit was lower in 2020-2021, with RRs of 0.83 (95% CI 0.76-0.90) in unadjusted analyses and 0.80 (95% CI 0.71-0.90) after adjusting for demographics, VL, and CD4. There was also a significant unadjusted decrease for 2020-2021 vs. 2017-2019 among women, men, PWH who were Black, White, < 40 or 50-59 years old, and with CD4 >500 (Fig. *B-F*, all *P*< 0.05).

**Figure:**
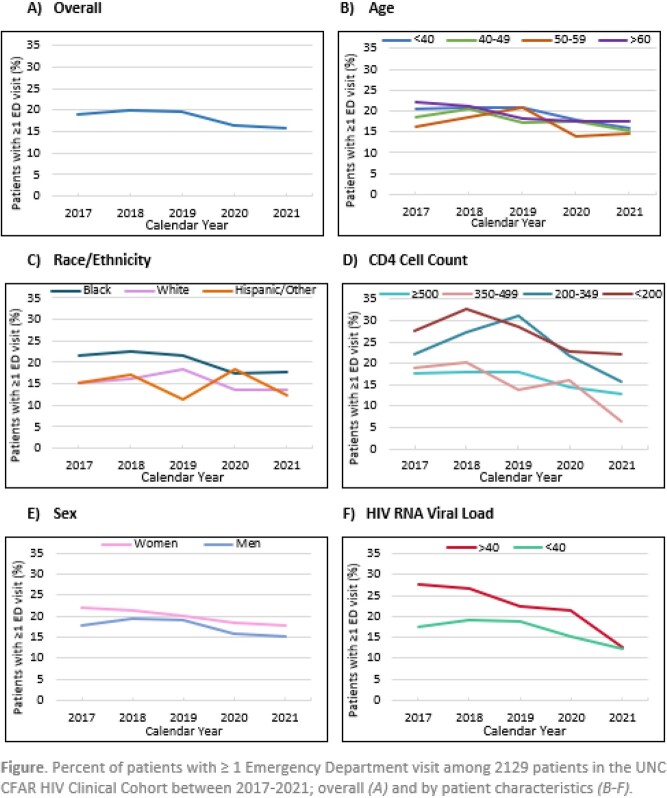

**Conclusion:**

Among PWH in HIV care, ED use was higher among women, Black PWH, and PWH with poorly controlled HIV. ED use decreased 2020-2021 in most groups, indicating that PWH during the COVID-19 pandemic may be delaying seeking care for acute conditions, or accessing care in other ways. Work is ongoing to characterize reasons for ED visits across calendar years and examine the impact of reduced ED utilization among PWH.

**Disclosures:**

**Joseph J. Eron, MD**, Adagio Therapeutics: data safety monitoring committee|Gilead Sciences: Advisor/Consultant|Gilead Sciences: Grant/Research Support|Glaxo Smith Kline: Advisor/Consultant|Merck: Advisor/Consultant|ViiV Healthcare: Advisor/Consultant|ViiV Healthcare: Grant/Research Support.

